# Comparative single-cell immune responses in peripheral blood and lymph node of immunized SARS-CoV-2 challenged infant rhesus macaques

**DOI:** 10.3389/fimmu.2025.1599408

**Published:** 2025-09-15

**Authors:** Sasha Anronikov, Cameron Meikle, Emma C. Milligan, Han Chen, Nilanjan Mukherjee, Zach Bjornson, Brice Gaudillière, Sizun Jiang, Sallie R. Permar, Koen K. A. Van Rompay, Garry P. Nolan, Kristina De Paris, David R. McIlwain

**Affiliations:** ^1^ Department of Microbiology and Immunology, School of Medicine, Stanford University, Stanford, CA, United States; ^2^ Department of Microbiology and Immunology, School of Medicine, University of Nevada, Reno, Reno, NV, United States; ^3^ Department of Microbiology and Immunology, University of North Carolina, Chapel Hill, NC, United States; ^4^ Department of Anesthesiology, Perioperative & Pain Medicine, Stanford University, Stanford, CA, United States; ^5^ Center for Virology and Vaccine Research, Beth Israel Deaconess Medical Center and Harvard Medical School, Boston, MA, United States; ^6^ Department of Pediatrics, Weill Cornell Medicine, New York, NY, United States; ^7^ California National Primate Research Center, University of California, Davis, Davis, CA, United States; ^8^ Department of Pathology, School of Medicine, Stanford University, Stanford, CA, United States

**Keywords:** vaccine, SARS-CoV-2, rhesus macaques, delta variant, peripheral blood mononuclear cells, lymph node, COVID-19, CyTOF

## Abstract

**Introduction:**

A better understanding of post-exposure immune responses in vaccinated individuals, particularly infants, is needed.

**Methods:**

Using a rhesus macaque model, we compared recipients of mRNA- or protein-based SARS-CoV-2 vaccines administered in infancy with unvaccinated controls 7 days post-SARS-CoV-2 virus challenge. Mass cytometry profiling of peripheral blood mononuclear cells and dissociated mediastinal lymph node cells at 7 days post-challenge revealed tissue-specific differences between groups, representing a snapshot of immune activity at this point.

**Results:**

Vaccinated animals showed lower frequencies of activated CD8+ T cells in blood and lower levels of monocyte and B cell subsets in lymph nodes, aligning with lower viral loads and milder pathology. Plasmacytoid dendritic cells—commonly depleted in circulation during severe human COVID-19—were preserved in the blood of vaccinated groups. *Ex vivo* stimulation demonstrated heightened inflammatory cell signaling from unvaccinated rhesus macaques, correlating with worse clinical outcomes.

**Discussion:**

These findings enhance our understanding of a critical nonhuman primate model and underscore the utility of single-cell, tissue-level analyses in evaluating next-generation pediatric SARS-CoV-2 vaccine strategies.

## Introduction

SARS-CoV-2, the virus responsible for the COVID - 19 pandemic, continues to have a significant impact on global health. Although children are generally less likely to experience severe COVID - 19 than adults (1–3), they are still capable of developing severe disease, facing long-term sequelae, and contributing to viral transmission (4, 5). With the emergence of new viral variants, it is increasingly important to establish and maintain effective vaccination strategies that include all age groups, especially infants and children. Currently, CDC vaccination recommendations apply only to individuals 6 months and older, partly because the understanding of vaccine responses and protection for infant immunization remains incomplete.

Rhesus macaques serve as a critical model for studying immune responses during vaccine development and licensure. This is because their immune systems and physiology share significant similarities with those of humans (6) and are susceptible to many of the same pathogens that affect humans, allowing for insights into vaccine-induced responses and protection.

Macaque challenge models have been pivotal in advancing our understanding of SARS-CoV-2. They have elucidated key aspects of the virus’s pathogenesis and cellular immune responses (7, 8), as well as played a crucial role in vaccine evaluation and the identification of correlates of protection (9–11). A deeper understanding of how these critical models respond to vaccination and challenge and their similarities and differences to humans remains a priority for developing next-generation SARS-CoV-2 vaccines. Infant macaques are particularly relevant for understanding how vaccines may function in infant populations, where key immune responses may differ from those of adults.

This study employs a comparative approach to evaluate the immune responses elicited after SARS-CoV-2 challenge in rhesus macaques that were immunized as infants with two different vaccine platforms: mRNA-based vaccines and protein-based vaccines, relative to an unvaccinated control group. Both platforms have demonstrated efficacy in prior reports of this study (12, 13). However, whether both types of vaccines result in similar cellular immune responses post-SARS-CoV-2 viral challenge remains less understood. Given that current SARS-CoV-2 vaccine strategies tend to have enduring protection against severe disease rather than long-term sterilizing immunity (14), a deeper understanding of the immune responses triggered in the post-exposure period in vaccinated individuals is warranted.

Mass cytometry (CyTOF) quantifies numerous phenotypic and functional markers at the single-cell level for millions of cells (15), enabling comprehensive characterization of immune subsets, activation states, and signaling capacities across tissues. We previously developed universal cross-species mass cytometry antibody panels for both human and non-human primate immune cells (6) and established workflows to apply these tools to gain insight from viral challenge studies ranging from high-containment non-human primate (NHP) studies (16) to human volunteer viral challenge settings (17, 18). Short-term *ex vivo* stimulation of immune cells can predict clinical inflammatory outcomes (19–23). Using a mass cytometry pipeline, we previously linked differences in immune cell frequencies and signaling capacities to COVID - 19 severity in humans (24). However, these effects have yet to be fully explored in NHP models.

Immune responses to viral infection can vary across different tissues and organs. While peripheral blood provides a readily accessible window into systemic immune responses, it may not fully capture immune reactions occurring in lymphoid tissues, where critical aspects of adaptive immunity are orchestrated. Moreover, mediastinal lymph nodes filter lymph fluid from the lung, a main organ target of SARS-CoV-2, and lymphadenopathy of the mediastinal lymph nodes is a known predictor of SARS-CoV-2 mortality in humans (25). This study examined immune responses in both peripheral blood and mediastinal lymph nodes, aiming to identify tissue-specific differences and gain a more comprehensive understanding of immune responses triggered after exposure to the SARS-CoV-2 virus in vaccinated and unvaccinated animals.

Mass cytometry was performed on dissociated lymph node cells and peripheral blood immune cells collected post-SARS-CoV-2 challenge from both infant-vaccinated and unvaccinated control rhesus macaques. This approach quantified cell population frequencies and assessed the capacity of these cells to generate signaling responses to *ex vivo* immune-activating stimuli. Our findings expand our understanding of this animal model and reveal features that closely resemble those observed in human COVID - 19 infection.

## Materials and methods

### Materials

### Methods

#### Immunization and challenge study

The immunization and challenge study has been previously described (12, 13). A total of 24 rhesus macaques of Indian origin from the California National Primate Research Center breeding colony were used. Median age 2.2-month-old male (n=8) and female (n=8) animals were randomly assigned into one of two vaccination groups, and another n=8 age- and sex-matched rhesus macaques were later added for a third unimmunized control group (mock). Animal care followed the *Guide for Care and Use of Laboratory Animals* by the Institute for Laboratory Animal Research. All animal procedures were approved by the University of California (UC) Davis Institutional Animal Care and Use Committee before study initiation.

Protein vaccine group infant animals received intramuscular doses of 15 mcg of the Washington strain S - 2P protein mixed with 10 mcg of 3M - 052 (in 2% squalene-in water emulsion) in a 0.5 mL volume divided across the right and left quadriceps at week 0 and biceps at week 4. S - 2P Protein was provided by The Vaccine Research Center (National Institutes of Health). 3M - 052-SE adjuvant was provided by the Access to Advanced Health Institute (AAHI) and 3M. mRNA vaccine group infant animals received intramuscular doses of 30 mcg of the Washington strain S - 2P SARS-CoV-2 mRNA-LNP at week 0 in the quadriceps and week 4 in the bicep. The SARS-CoV-2 mRNA-LNP was provided by Moderna, Inc.

All groups were challenged at 52 weeks post-first immunization with a Delta variant (Lineage B.1.617.2) challenge strain administered through combined intratracheal (2x10^6^ pfu in 1 mL PBS solution) and intranasal (1X10^6^ pfu, 0.25 mL PBS solution per nostril) routes. Animals were euthanized on day 7 post-challenge.

#### Blood sample collection

Blood was collected by peripheral venipuncture on day 7 post-challenge. PBMCs were processed from whole blood, counted, and viably frozen in 1mL aliquots containing 10x10^6^ cells in heat-inactivated sterile-filtered FBS containing 10% DMSO and stored in a liquid nitrogen freezer. Complete blood counts were performed and previously reported by Milligan et al. (13).

#### Lymph node sample collection

A full necropsy was performed after euthanasia on day 7 post-challenge for tissue collection. Mediastinal lymph nodes were trimmed of connective tissue and fat, mechanically disrupted in PBS, filtered through a 40 µm filter, centrifuged at 1800 rpm for 10 minutes, washed with PBS, and counted. Dissociated mediastinal lymph node cells were viably frozen in 1 mL aliquots containing 10×10^6^ cells in heat-inactivated, sterile-filtered FBS with 10% DMSO and stored in a liquid nitrogen freezer. Lymph node weight and cellularity were not determined.

#### Cell stimulation

Cryopreserved PBMC and dissociated LN samples were thawed in PBS containing benzonase, washed two times, counted, and resuspended to a concentration of 4x10^6^ cells per mL, and rested at 37°C for at least 30 minutes. Approximately 3x10^6^ cells were then stimulated by incubation for 15 minutes at 37°C with PMA/Ionomycin (1X Cell Stimulation Cocktail), 10 mcg/mL Resiquimod (R848) (in VivoGen), or PBS (unstimulated control). After stimulation, samples were fixed in a final concentration of 1.6% PFA for 10 minutes at room temperature. Fixed samples were centrifuged, and pellets were resuspended in cell staining medium (PBS + 0.05% BSA) and stored frozen at -80°C. Functional validation of the assay was confirmed in a pilot set of study samples by demonstrating induction of expected phospho-signaling readouts. Viability staining was not incorporated into the assay.

#### Cell staining and mass cytometry data acquisition

Frozen fixed stimulated PBMC and LN suspension were thawed, resuspended in cell staining medium (PBS + 0.5% BSA + 0.02% sodium azide), and placed in a 96-well block for barcoding and staining using a robotic staining platform (6). Sets of 16 samples were barcoded using palladium metal isotopes and pooled into a single well as previously described (26). Pooled, barcoded samples were incubated with Fc-block (Human TruStain FcX, Biolegend) for 10 minutes before 30-minute incubation with a lyophilized cocktail of metal-tagged cell surface antibodies in cell staining medium. After surface staining, cells were permeabilized with ice-cold 100% methanol, washed, and stained for 60 minutes with a lyophilized metal-tagged intracellular antibody cocktail in cell staining medium (see **Table 1** and **Supplementary Table S1** for the antibody panel and concentrations). Stained cells were then washed and resuspended in an iridium intercalator (Standard Biotools) solution containing 1.6% paraformaldehyde. Finally, the samples were washed, resuspended in 1X five-element normalization beads (Standard Biotools), and analyzed on a freshly cleaned and tuned CyTOF2 instrument (Standard Biotools).

**Table 1:** Key resources.

Reagent or resource	Source	Identifier
Antibodies		
CD233 (BRIC 6)	IBGRL	Cat# 9439; RRID: AB_3676420
CD45 (DO58 - 1283)	BD Biosciences	Cat# 552566; RRID: AB_394433
CD61 (VI-PL2)	Biolegend	Cat# 336402, RRID: AB_1227584
CD7 (M-T701)	BD Biosciences	Cat# 555359, RRID: AB_395762
CD33 (AC104.3E3)	Miltenyi	Cat# 130 - 108-039, RRID: AB_2660358
CD11c (3.9)	Biolegend	Cat# 301602, RRID: AB_314172
CD123 (7G3)	BD Biosciences	Cat# 554527, RRID: AB_395455
CD14 (M5E2)	Biolegend	Cat# 301810, RRID: AB_314192
CD11b (ICRF44)	Biolegend	Cat# 301312, RRID: AB_314164
CD8 (RPA-T8)	Biolegend	Cat# 301002, RRID: AB_314120
CD4* (OKT4)	Biolegend	Cat# 317404, RRID: AB_571961
CD3 (SP34.2)	BD Biosciences	Cat# 551916, RRID: AB_394293
CD66 (YTH71.3)	Thermo Fisher	Cat# MA5 - 17003, RRID: AB_2538475
CD16 (3G8)	Biolegend	Cat# 302033, RRID: AB_2104002
CD1c (AD5 - 8E7)	Miltenyi	Cat# 130 - 108-032, RRID: AB_2661165
BDCA3 (1A4)	BD Biosciences	Cat# 559780, RRID: AB_397321
CD45RA (HI100)	Biolegend	Cat# 304102, RRID: AB_314406
CD161 (HP-G310)	Biolegend	Cat# 339902, RRID: AB_1501090
FITC (FIT - 22)	Biolegend	Cat# 408302, RRID: AB_528901
CD20 (2H7)	Biolegend	Cat# 302302, RRID: AB_314250
IgM (G20 - 127)	BD Biosciences	Cat# 555780, RRID: AB_396115
CD56 (NCAM16.2)	BD Biosciences	Cat# 559043, RRID: AB_397180
HLA-DR (Immu357)	Beckman Coulter	N/A
CCR7 (150503)	BD Biosciences	Cat# 561271, RRID: AB_10561679
STAT1 pY701 (4a)	BD Biosciences	Cat# 612233, RRID: AB_399556
STAT3 pY705 (4)	BD Biosciences	Cat# 612357, RRID: AB_399646
STAT4 pY693 (38)	BD Biosciences	Cat# 612738, RRID: AB_399957
STAT5 pY694 (46)	BD Biosciences	Cat# 611965, RRID: AB_399386
STAT6 pY691 (18, J71 - 773.58.11)	BD Biosciences	Cat# 611597, RRID: AB_399039
Ki67 (SolA15)	Thermo Fisher	Cat# 14 - 5698-82, RRID: AB_10854564
Erk1/2 pT202/Y204 (D13.14.4E)	CST	Cat# 4370, RRID: AB_2315112
MAPKAPK2 pT334 (27B7)	CST	Cat# 3007, RRID: AB_490936
CREB pS133 (87G3)	CST	Cat# 9198, RRID: AB_2561044
IkBa amino-terminal (L35A5)	CST	Cat# 4814, RRID: AB_390781
TBK1/NAK pS172 (D52C2)	CST	Cat# 5483, RRID: AB_10693472
S6 pS235/236 (2F9)	CST	Cat# 4856, RRID: AB_2181037
Zap70/Syk pY319/Y352 (17a)	BD Biosciences	(BD Biosciences Cat# 612574, RRID: AB_399863)
4E-BP1 pT37/46 (236B4)	CST	Cat# 2855, RRID: AB_560835
PLCγ2 pY759 (K86 - 689.37)	BD Biosciences	Custom
P38 pT180/Y182 (36/p38)	BD Biosciences	Cat# 612289, RRID: AB_399606
FITC (for CCR7) (FIT - 22)	Biolegend	Cat# 408302, RRID: AB_528901
FoxP3 (PCH101, NRRF - 30)	Thermo Fisher	Cat# 14 - 4776-82, RRID: AB_467554
Chemicals, peptides, and recombinant proteins		
Fetal Bovine Serum (FBS)	Fisher Scientific	Cat# Gibco 16140071
R848	Sigma Aldrich	Cat# SML0196 - 10MG
PI	Invitrogen	Cat# Fisher 00497503
Bovine Serum Albumin (BSA)	Sigma Aldrich	Cat# A3059 - 50G
Sodium Azide	Sigma Aldrich	Cat# S2002 - 25G
Critical commercial assays		
FcBlock: Human TruStain FcX	Biolegend	Cat# 422302; RRID: AB_2818986
100% Methanol	Thermo Fisher	Cat# 50 - 980-487
Iridium DNA Intercalator	Fluidigm	Cat# 201192B
16% Paraformaldehyde	Thermo Fisher	Cat# 50 - 980-487
Four Element Normalization Beads	Fluidigm	Cat# 201078
Deposited data		
Raw and processed data	Mendeley	https://data.mendeley.com/preview/w7685x343j?a=0e265441-8196-4730-b566-bdb6b6111e16
Software and algorithms		
CellEngine	CellCarta	https://cellengine.com
Single Cell Debarcoder	Nolan Lab	https://github.com/nolanlab/single-cell-debarcoder
Bead Normalization	Nolan Lab	https://github.com/nolanlab/bead-normalization
R/RStudio	Posit	https://posit.co/download/rstudio-desktop/

#### Normalization and debarcoding

To minimize technical variation, collected data were first normalized across all barcode batches using values from normalization beads that were spiked into samples, using the normalization protocol established by Finck et al. (27) After normalization, a single-cell debarcoding algorithm (26) separated the data into individual sample FCS files by applying Mahalanobis distance intensity thresholds across palladium barcode metal channels.

#### Gating, calculation of cell frequency, pseudo-absolute counts, and signaling marker levels

Normalized and debarcoded FCS files were analyzed and visualized using CellEngine. Cell populations were manually gated and annotated (see **Supplementary Figure S1** for gating strategy). Cell population frequencies were calculated as the percentage of mononuclear (CD45^+^CD66^-^) cells. To approximate absolute cell counts for peripheral blood populations, complete blood count (CBC) data were used to calculate absolute mononuclear cell counts as: WBC count – (neutrophil count + eosinophil count). This CBC-derived mononuclear fraction was assumed to correspond to the same CD45^+^CD66^-^ mononuclear fraction defined by CyTOF, and these values were multiplied by the corresponding population frequencies to generate ‘pseudo-absolute counts’, expressed as cells/µL of blood (see **Supplementary Table S2**). All frequency and ‘pseudo-absolute count’ comparisons were made using cells from the unstimulated condition. Cell population signaling marker intensities were calculated using arcsinh-transformed median signal intensity values (arcsinh(x/5)) for each population under each stimulation condition. Stimulation-dependent signaling events were examined by comparing signaling marker intensities between unstimulated and stimulated cells from the same population.

#### Statistical analysis in R

Graphing and statistical analysis were performed using R. All code used for this paper is freely available on Github (https://github.com/sasha-anronikov/CIV-CyTOF). Packages used: dplyr, tidyverse, ggplot2, ggpubr, ggdendro, grid, gridExtra, svglite, patchwork, ggpattern, tidyselect, stats, ggalluvial, reshape2, ggrepel. Tests between multiple groups were performed using an ANOVA. Tests between pairs of groups were performed using a student’s t-test. Correlations were computed as a Pearson correlation. All statistical tests were controlled for multiple hypotheses with the Benjamini-Hochberg procedure unless otherwise stated.

#### UMAP

UMAPs were generated and visualized on CellEngine using unstimulated cell data and the following phenotypic markers: CD38 (Y89Di), CD7 (Pr141Di), CD123 (Nd148Di), CD11b (Eu153Di), CD8 (Gd155Di), CD4 (Gd156Di), CD3 (Gd157Di), CD66 (Gd158Di), CD16 (Tb159Di), CD19 (Yb173Di), IgM (Yb174Di), CD56 (Lu175Di), HLA-DR (Yb176Di). Subsampling was performed to 15,000 cells per sample, iterated over 200 times, with n-neighbors = 40 and minimum distance = 0.1.

#### Box plots

Boxplots were generated in R to visualize the distribution of cell population frequencies across tissue types (PBMCs and LN) and vaccine groups (Mock, mRNA, and Protein). Boxplots were created using the ggplot2 package, displaying the median, interquartile range, and whiskers to denote variability outside the upper and lower quartiles.

#### Alluvial plot

An alluvial plot visualizes the distribution of cell population frequencies across tissue types (PBMCs and LN) and vaccine groups (Mock, mRNA, and Protein). Frequencies were averaged and corrected to account for nested subpopulations (e.g., subtracting ‘B cell IgM+’ frequencies from their parent total ‘B cell’ frequencies). Plots were generated using the ggalluvial and ggplot2 packages in R. The flow of the alluvial plot represents the mean frequencies of each cell population, while the strata correspond to tissue type, vaccine group, and cell population.

#### Correlation plots

Custom scatter plots were created to compare cell population frequencies between PBMCs and LN across vaccine groups (Mock, mRNA, and Protein). Unstimulated cell frequencies were averaged for each vaccine group within PB and LN. The axes, displaying PBMC and LN frequencies, were log-scaled to enhance the visualization of a wider range of frequency differences. For cell populations that exhibited significant differences in frequency between mock and vaccine groups in either tissue type (T-test, Benjamini-Hochberg adjusted p <0.05), values were highlighted by connecting the three associated vaccine group data points with a shaded triangle. Plots were generated using ggplot2 and ggrepel in R. Scatter plots were also created to compare clinical outcome data with cell population frequencies and cell signaling features using R with the ggplot2, ggpubr, dplyr, and tidyverse packages. Statistical analysis was performed using Pearson’s method and corrected with the Benjamini-Hochberg procedure.

#### Volcano plots

Volcano plots were created in R using the ggplot2 and ggpubr packages. Plots display cell population-signaling marker features, mean post-stimulation intensity difference, and uncorrected T-test p-value. Features meeting p<0.05 and mean magnitude of change >0.1 or <−0.1 are colored red (upregulated) or blue (downregulated), respectively. The top five upregulated and downregulated features determined by the combined ranking of p-value and change in mean feature for each volcano plot are labeled.

#### Heatmaps

Heatmaps were generated in R using the ggplot2, ggdendro, and ggpattern packages. Population frequency heatmaps display the log2 ratio of the vaccine group population frequency relative to the mock control. For signaling analysis, the top 20% of cell population × signaling marker features for all animals pooled regardless of immunization status—ranked by both p-value and magnitude of post-stimulation change, were assessed by ANOVA to identify potential differences between vaccine groups. The top three cell-type-signaling marker features differing between vaccine groups (by ANOVA) for each stimulation condition and tissue type were plotted in a heatmap. The heatmap color scale indicates the Z-score, calculated as the number of standard deviations by which a vaccine group’s mean value differs from the overall mean for that specific cell type–signaling marker feature. The size of each tile corresponds to the Benjamini–Hochberg corrected significance values from the ANOVA test for differences between vaccine groups. For heatmaps illustrating correlations between cell population frequencies or top cell signaling features and clinical outcomes, the color scale represents Pearson’s R values, and crosshatching denotes cases where the Benjamini–Hochberg adjusted p-value is greater than 0.05. R848 pDC response heatmaps were created using CellEngine and show median channel levels (R848 – unstimulated).

#### Outcome and clinical data

The clinical outcome data used were previously published (13). SARS-CoV-2 RNA corresponds to the *N gene* (log_10_ copies/30 mg of lung tissue) as determined by qPCR from day 7 necropsy tissue. Lung tissue pathology scores, from 0 to 4, were determined by the extent and severity of the interstitial and alveolar inflammation in day 7 necropsy lung tissue, with a greater score corresponding to greater damage. Lung radiographs from day 7 were scored by a board-certified veterinary radiologist using a standard scoring system from 0 to 3. An increasing score corresponds to higher lung damage and interstitial infiltrates.

## Results

### Study design and mass cytometry pipeline

**Figure 1:**
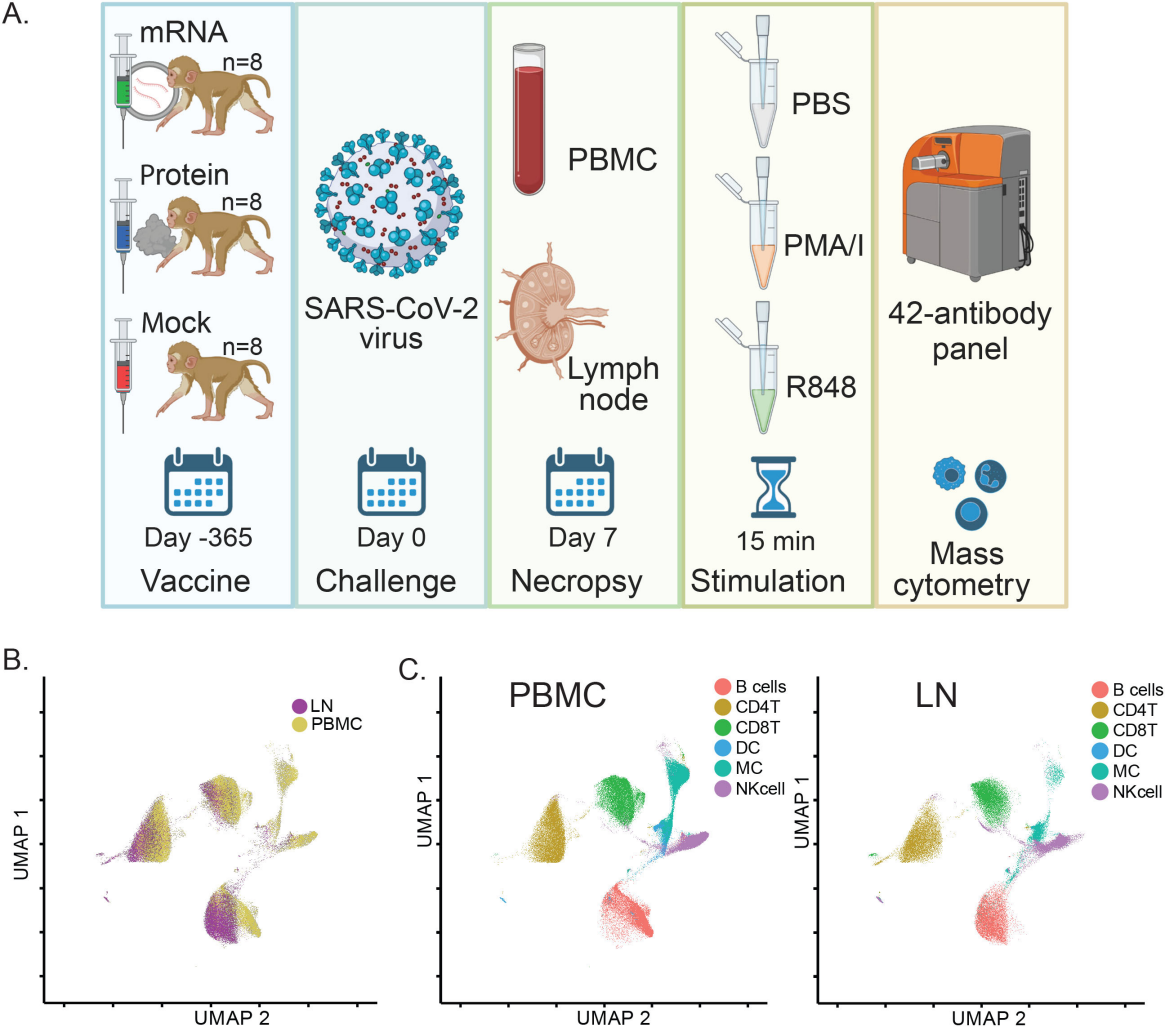
SARS-CoV-2 vaccination and challenge study design overview and assessment of matched immune cell populations in peripheral blood and lymph nodes. **(A)** Study Schematic: Infant rhesus macaques were immunized against SARS-CoV-2, with 8 receiving an mRNA-LNP vaccine and 8 receiving a protein-based S - 2P Protein+3M052-SE vaccine. Eight additional animals received a mock vaccination. At 52 weeks post-vaccination, all animals were challenged with the SARS-CoV-2 Delta variant. PBMC and LN samples were collected 7 days post-challenge during necropsy, treated with PBS (unstimulated control), PMA/Ionomycin, or R848 for 15 minutes, and analyzed using a 42-antibody mass cytometry panel to identify cellular populations and signaling marker levels. **(B)** A global UMAP visualization of mass cytometry data showing all mononuclear (CD45^+^CD66^-^) cells for unstimulated conditions colored by tissue source. **(C)** Individual UMAPs for the same unstimulated cells shown in **(B)** are separated by tissue source (blood, left panel; lymph node, right panel). Cells are colored by assigned annotated cell populations.

As part of a previously described SARS-CoV-2 vaccination and challenge study, a total of n=24 infant macaques were assigned to treatment groups receiving a prime and 4-week boost of either a SARS-CoV-2 mRNA-Lipid Nanoparticle (LNP) intramuscular vaccine (mRNA, n=8), an adjuvanted protein-based SARS-CoV-2 intramuscular vaccine (S - 2P Protein+3M-052 SE) (Protein, n=8), or non-immunized control (Mock, n=8) (12, 13) (**Figure 1A**). Animals from all three groups were challenged 52 weeks post-first immunization with a combined intratracheal and intranasal administration of a SARS-CoV-2 Delta variant (Lineage B.1.617.2) strain. This variant was chosen for challenge because it was the dominant circulating strain at the time, allowing assessment of protection against a heterologous virus. The 52-week interval between immunization and challenge was selected to test the durability of immune responses. In this cohort of macaques, prior vaccination and SARS-CoV-2-neutralizing and binding antibodies were associated with faster viral clearance and protection from clinical signs and lung pathology post-challenge (13).

To examine tissue-specific single-cell immune responses in immunized SARS-CoV-2 challenged rhesus macaques from this study, we used mass cytometry to profile peripheral blood mononuclear cells (PBMC) and dissociated mediastinal lymph node (LN) cells isolated from animals sacrificed and necropsied at 7 days post-challenge. To assess both immune cell phenotype and immune signaling responses, cryopreserved PBMC and LN cells were thawed and stimulated *ex vivo* with PMA/Ionomycin (PMA/I), R848, or PBS (unstimulated control) for 15 minutes before fixation. Fixed, stimulated cells were then barcoded with palladium metals (26) and stained with a panel of 42 metal-tagged antibodies recognizing phenotypic cell surface markers, activation markers, and phospho-specific epitopes of signaling markers (**Supplementary Table S1**) (6).

Samples were analyzed using a CyTOF mass cytometer, generating single-cell data for a total of 20 million LN cells and 38 million PBMCs. Data were normalized to bead standards and debarcoded using an algorithm that removes cell-cell doublets (26).

Using phenotypic cell surface markers, we created UMAP embeddings incorporating data from both tissue types for unstimulated samples. This resulted in substantial overlap and confirmed that similar cell types were quantifiable in both PBMC and LN samples (**Figure 1B**). This approach was complemented by a universal gating strategy (**Supplementary Figure S1A**) to annotate matching immune cell populations in each tissue, which similarly showed consistent localization across tissues when projected onto UMAPs (**Figure 1C**).

To ensure technical consistency, repeated control samples were barcoded, pooled, stained, and run alongside batches of study samples. These repeated controls showed uniform distribution and proportions of immune cell populations between run batches (**Supplementary Figure S2A**). Furthermore, as expected, proportions of immune cell populations were also consistent between samples treated with the different *ex vivo* stimulations used to trigger immune cell signaling (**Supplementary Figure S2C**).

Taken together, these data demonstrate the successful application of a mass cytometry pipeline across multiple tissues to generate data for comparisons of immune cell population frequencies, activation states, and signaling responses among different vaccine groups in rhesus macaques.

### Vaccination causes differences in immune cell population frequencies post-challenge

**Figure 2:**
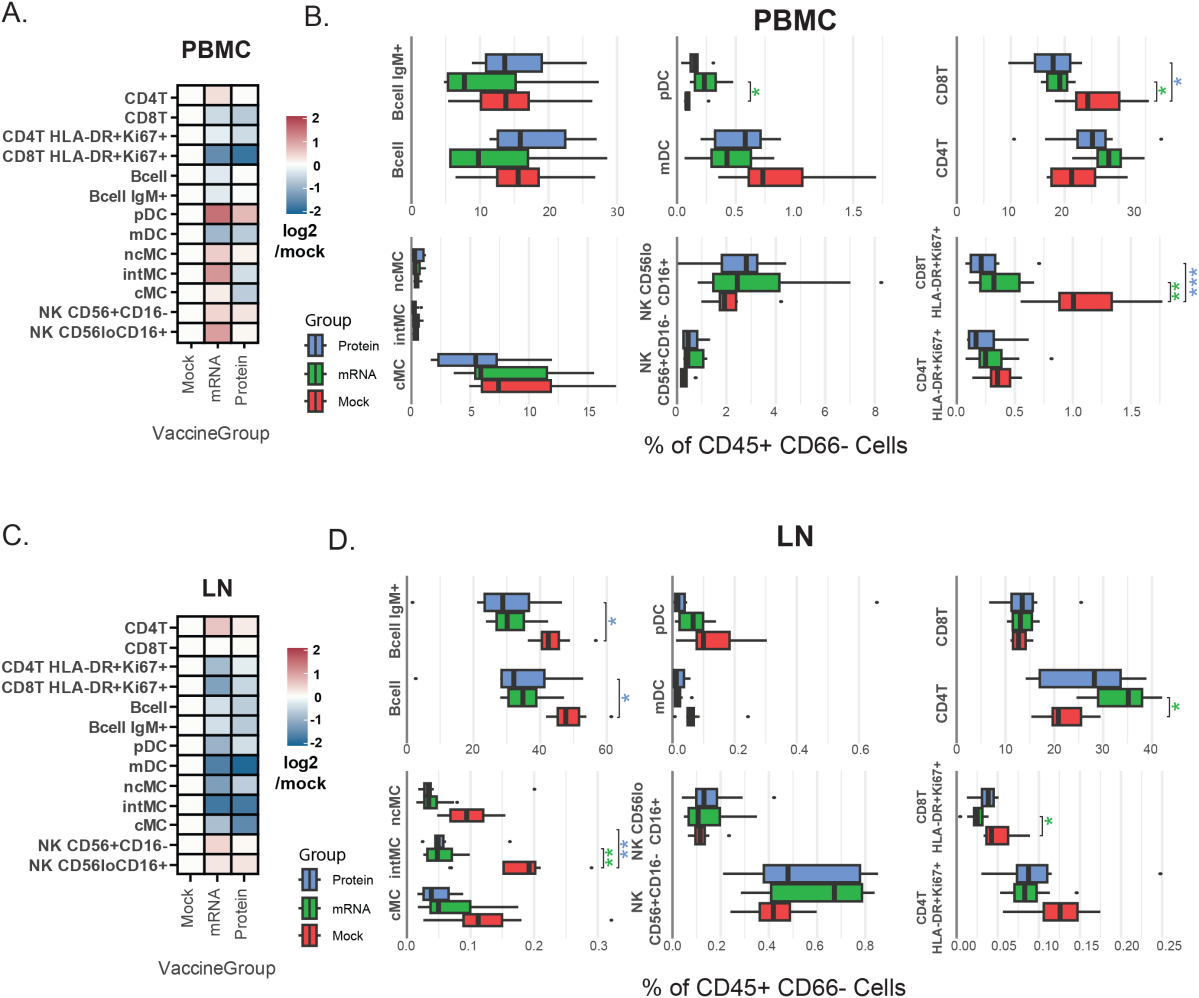
Vaccine group-specific variations in immune cell population frequencies. **(A, C)** Heatmaps representing annotated cellular population frequencies (rows) by vaccination group (columns) in PBMCs **(A)** and LN **(C)**. The color scale indicates the mean frequency (% of CD45^+^CD66^-^ cells) relative to the mock group, calculated as log_2_(vaccination group/mock). A positive log_2_ value indicates a higher frequency in the vaccinated group compared to the mock group, while a negative value indicates a lower frequency. **(B, D)** Box plots showing cell population frequencies (% of CD45^+^CD66^-^ cells) by vaccination group in PBMCs **(B)** and LN **(D)**. These box plots provide the underlying data and statistical comparisons for the trends shown in the heatmaps. Asterisks indicate Benjamini–Hochberg-adjusted significance levels from a Student’s t-test comparing each vaccination group to the mock group (*p<0.05, **p<0.005, ***p<0.001). The color of the asterisks denotes the vaccination group being compared (green = mRNA, blue = protein). Cell population abbreviations: CD4+ T cell (CD4T), CD8+ T cell (CD8T), plasmacytoid dendritic cell (pDC), myeloid dendritic cell (mDC), monocyte (MC) [classical: cMC, non-classical: ncMC, intermediate: intMC].

Frequencies of 13 myeloid and lymphoid cell populations were quantified at day 7 post-vaccination in PBMC and LN samples by calculating cell population abundance as a percent of total mononuclear (CD45+CD66-) cells, providing a snapshot of immune composition at this stage. Data were explored using heatmaps for log-fold differences in population frequencies between mRNA and protein vaccine groups relative to the mock vaccine control for peripheral blood (**Figure 2A**) and LN (**Figure 2C**).

ANOVA determined overall differences between groups, and t-test determined specific differences between vaccine groups and mock vaccine control. Benjamini-Hochberg correction was applied to adjust p-values to account for multiple hypotheses (**Supplementary Table S2**). Asterisks next to box plots indicate the significance level of these differences for PBMC (**Figure 2B**) and LN (**Figure 2D**) samples.

Notably, in peripheral blood, levels of activated CD8+ T cells (HLA-DR+Ki67+) were higher in mock-vaccinated animals versus either mRNA or protein-vaccinated animals (**Figure 2B**), consistent with higher viral loads observed in this group (13). Furthermore, blood plasmacytoid dendritic cells (pDC) levels were lower in mock-vaccinated animals than in mRNA-vaccinated animals, with a similar (but not significant) trend observed when comparing mock-vaccinated and protein-vaccinated animals (**Figure 2B**). These results are also consistent with reports of decreased pDC frequency during severe human SARS-CoV-2 infections (24, 28–32).

In LN samples, on the other hand, the most prominent differences between vaccine groups were elevated monocyte and B cell subsets in the mock-vaccinated group. Specifically, significantly higher frequencies of both total B cells and IgM+ B cells, as well as intermediate (CD11b+CD16+) monocytes, were observed in LN samples from mock-vaccine relative to mRNA and protein vaccine group animals (**Figure 2D**).

For peripheral blood, although absolute cell counts were not performed, complete blood count (CBC) data are provided and were additionally used to approximate cellularity (‘pseudo-absolute counts’) from population frequency data, in a parallel analysis to that described above for frequencies, with similar results (see Methods and **Supplementary Table S2**).

To directly visualize how post-SARS-CoV-2 challenge immune responses compared between tissues, calculated frequencies of all 13 quantified immune cell populations were plotted as the mean proportion of immune cells in LN versus PBMC using an alluvial plot (**Supplementary Figure S3**). In this plot, the width of each flow represents the mean frequency of a given immune cell population, and the flows connect cell populations across tissue types and vaccine groups to highlight their relative abundance across compartments. This analysis, and population frequency values reported in **Supplementary Table S2**, emphasize that observed cell type frequencies align with the expected distinct composition of these two immune tissues; for instance, lymph nodes—primary sites of germinal centers—harbor a higher frequency of IgM+ B cells than peripheral blood (33).

**Figure 3:**
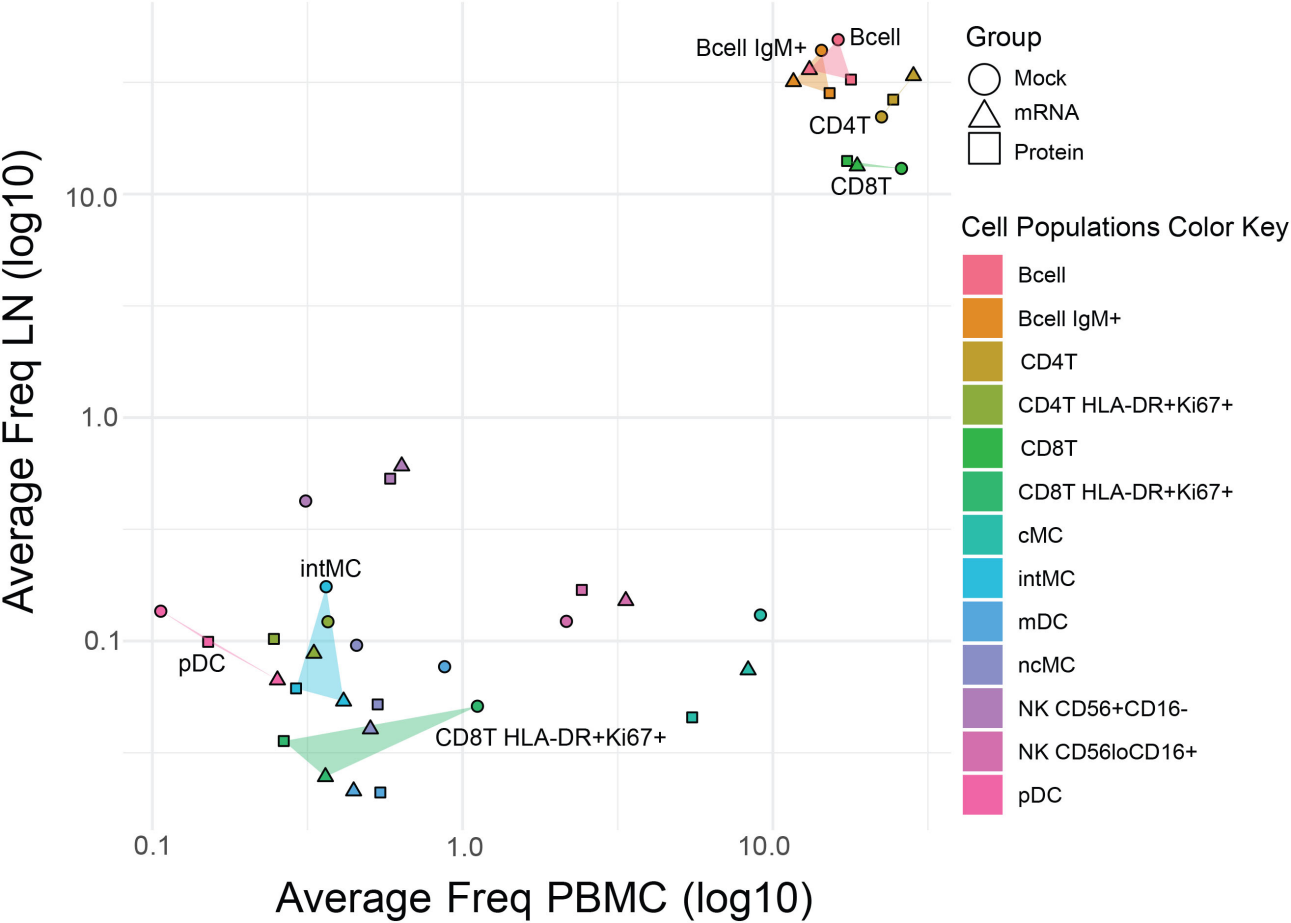
Comparative analysis of immune cell frequencies in lymph nodes and peripheral blood across vaccine groups. Scatter plot showing the mean frequency of each immune cell population in PBMCs (x-axis) versus LNs (y-axis) to assess the consistency effects of vaccination post-virus challenge across tissues. Points represent vaccine groups (Mock: circles, mRNA: triangles, Protein: squares), with cell population identity indicated by the color key. For cell populations that differed significantly in frequency between mock and vaccine groups in either tissue (see **Figure 2**), the area between data points is shaded (translucent triangles) for easier visualization. Highlighted populations include pDC, intMC, CD8^+^ T HLA-DR^+^Ki67^+^, B cells, IgM^+^ B cells, CD4^+^ T cells, and CD8^+^ T cells.

An annotated biaxial plot was also constructed to correlate immune cell population frequencies between tissues (**Figure 3**). Post-SARS-CoV-2 challenge, LN and PBMC immune cell population frequencies were strongly correlated overall (r=0.78, p=5.6x10^-9^; Pearson) as well as within individual vaccine treatment groups (mRNA r=0.81, p=8.3x10^-4^; protein r=0.92, p=1.1x10^-5^; mock r=0.67, p=1.2x10^-2^; Pearson). Shaded regions on the biaxial plot highlight populations with significantly divergent frequencies (p<0.05, T-test **Supplementary Table S2**) between vaccine groups in either LN or PBMC, revealing tissue-specific patterns in vaccine-dependent responses following SARS-CoV-2 challenge (**Figure 3**). For instance, when comparing mock-vaccinated versus vaccinated animals, increased activated CD8+ T cells (HLA-DR+Ki67+) were evident in both tissues, but were much more pronounced in the blood. pDC levels, however, were lower in mock-treated animals compared with vaccinated groups in PBMC but not in LN. Whereas, elevations in intermediate monocytes (intMC) and B cell subsets were confined only to mock-vaccinated LN (**Figures 2**, **3**).

Compared to the mock control, vaccination with either mRNA or protein vaccines resulted in significant differences in post-virus challenge cell population frequencies in rhesus macaques, which were largely tissue-specific.

### Tissue-specific immune cell signaling patterns post-SARS-CoV-2 challenge

The capacity of immune cells to respond to short-term *ex vivo* stimuli can be predictive of various clinical inflammatory states (19–23); here, we assessed these signaling responses at a single time point (day 7 post-challenge) to capture the immune landscape present at this stage. We previously linked differences in such signaling capacities to the severity of COVID - 19 in humans (24), but these effects have not yet been explored in NHP models. Therefore, we examined immune signaling responses by measuring 15 intracellular signaling antibody markers in PBMC and LN cells stimulated *ex vivo* with PMA/I (to trigger calcium signaling), R848 (a TLR7/8 agonist), or PBS (unstimulated control).

**Figure 4:**
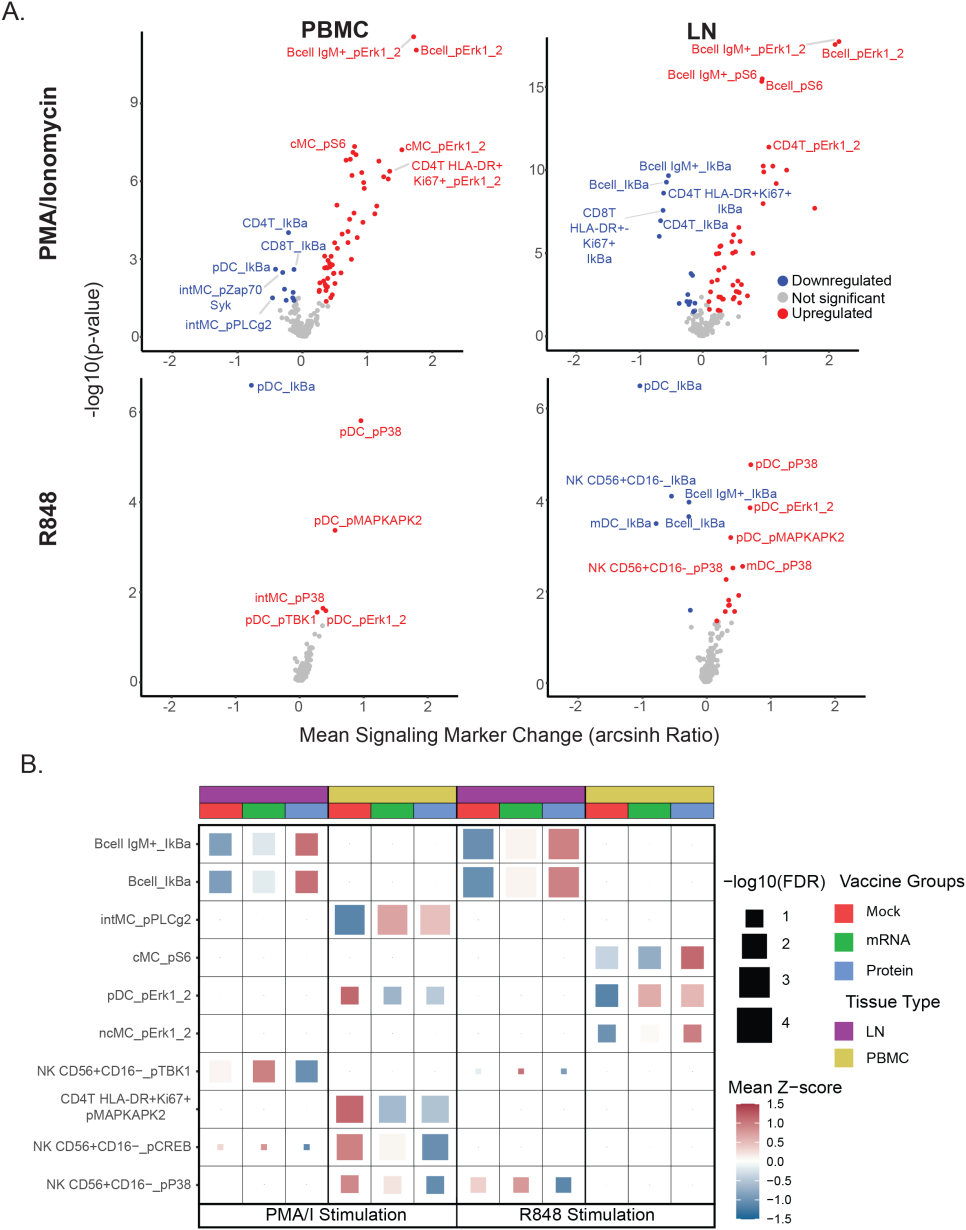
Vaccine group-specific differences in immune cell signaling responses. **(A)** Volcano plots display stimulation-dependent signaling responses across all immunization groups, comparing each stimulation condition (PMA/I or R848) with the unstimulated (PBS) control. Mean post-stimulation intensity differences (x-axis) and uncorrected t-test p-values (y-axis) are shown for each cell population–signaling marker feature. Features meeting p < 0.05 and mean arcsinh change > 0.1 or < −0.1 are colored red (upregulated) or blue (downregulated), respectively; the top five upregulated and downregulated features (by p-value and mean change) are labeled. This global analysis was used to rank all features, and the top 20% most responsive features (regardless of vaccine group) were carried forward for panel B analysis. **(B)** Heatmap of top cell population–signaling marker features differing by vaccine group. Each tile represents a feature for a given tissue, stimulation condition, and vaccine group, with tile color showing the mean Z-score (relative to the feature’s overall mean) and tile size indicating the multiple–hypothesis–corrected ANOVA significance. The top three features (by ANOVA) per tissue–stimulation condition are shown. See Materials and Methods for details.

Volcano plots were used to compare mean levels of signaling markers for each immune cell population and stimulation condition against unstimulated controls across all immunization groups, in order to identify the largest and most consistent changes induced post-stimulation at a global level as a prior step to analyzing differences between groups (**Figure 4A**). At a global level, PMA/I triggered more differentially regulated features than R848 for both PBMC and LN samples. B cells, T cells, and monocytes were the highest responders to PMA/I, whereas pDCs were the most responsive to R848. As expected, several phospho-targets—including pErk1/2, pS6, pP38, and pMAPKAP2—were significantly upregulated by either R848 or PMA/I, consistent with the ability of these stimuli to activate immune signaling pathways. Conversely, most significant downregulation events post-stimulation involved reductions in IkB, a signaling marker that is rapidly degraded following activation (34).

To look for possible differences in signaling capacity post-SARS-CoV-2 challenge related to prior immunization status, the top 20% of global cell populations x phospho-signaling marker features were first extracted for each stimulation condition and tissue type to identify the set of features with the highest responses to *ex vivo* stimulation to use for all subsequent analyses in this study. This feature set was used to examine potential differences in immune cell signaling between vaccine treatment groups by ANOVA with p-value adjustment using Benjamini-Hochberg correction (**Figure 4B**, **Supplementary Table S2**). Given the critical role of pDCs in early antiviral immunity and their depletion in severe COVID - 19 (24, 28–32), we examined their R848 responses to assess type I interferon–associated pathways (**Supplementary Figure S4**). R848 activated canonical TLR–MAPK/NF-κB markers in pDCs, with similar patterns in LN and PBMC. In PBMCs, pDCs from unvaccinated animals showed elevated pErk1/2 after PMA/I but reduced activation with R848 (**Figure 4B**). Notably, in LN, B cell subsets from unvaccinated challenged animals exhibited greater IκB downregulation from PMA/I and R848 stimulation, indicating enhanced NF-κB signaling compared to cells from vaccine-treated groups. In PBMCs, these same animals also showed heightened MAPK pathway activation (pMAPKAP2, pCREB, pP38) in lymphoid cells following PMA/I. Together, these data suggest that relative to vaccinated groups, heightened PMA/I signaling capacity, along with reduced pDC R848 responsiveness, may be a feature of unvaccinated animals post-challenge.

### Cell frequency and signaling responses correlate with post-challenge clinical outcomes

**Figure 5:**
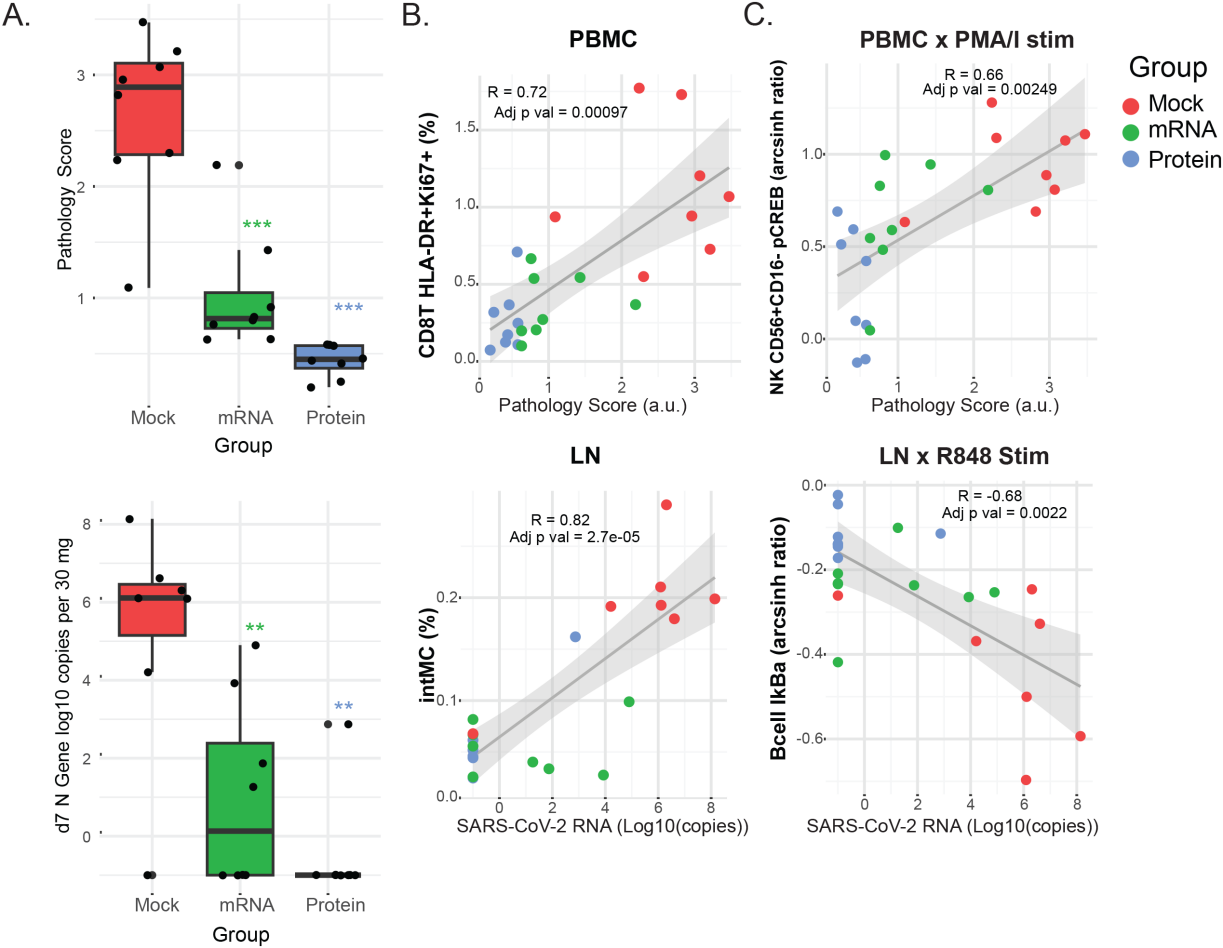
Clinical outcomes and their correlation with immune cell frequencies and signaling. **(A)** Box plots show the distribution of clinical outcome data for each vaccine group 7 days post-challenge. Lung pathology scores (0 - 4) from necropsy tissue and viral RNA copies (log10 copies per 30 mg of lung tissue) as determined by qPCR. Differences between the groups were determined by Mann-Whitney test with *P < 0.05, **P < 0.01, and ***P < 0.001 with FDR corrections. **(B, C)** Scatterplots showing the top correlations between either immune cell population frequencies **(B)** or cell signaling responses **(C)** and clinical outcomes, for PBMC (left panels) or LN (right panels) samples. Pearson’s correlation coefficients (R) and adjusted p-values are indicated within each plot. Linear regression lines and shaded 95% confidence intervals are overlaid. Dot color indicates the vaccine group: protein (blue), mRNA (green), or mock (red). For all panels: Cell frequencies are given as the percentage of mononuclear cells (CD45^+^CD66^-^). Cell signaling is shown as the arcsinh ratio of signaling marker levels relative to unstimulated controls for the indicated cell populations and stimulation conditions (PMA/I or R848). Pathology scores are lung tissue pathology scores from 0 to 4. SARS-CoV-2 RNA is measured as N gene (log_10_ copies per 30 mg of lung tissue).

Prior analyses from this study quantified viral shedding, clinical signs, and lung pathology following SARS-CoV-2 challenge, demonstrating protective effects for both vaccines, with the greatest protection observed in the protein vaccine group (**Figure 5A**) (13). Given the range of clinical outcomes observed, we next evaluated whether mass cytometry–derived cell frequencies and signaling features correlated with these outcomes. To do this, we calculated Pearson correlation coefficients (R) and Benjamini–Hochberg–adjusted p-values between the mass cytometry data and three continuous outcome variables: (1) quantitative SARS-CoV-2 viral RNA (qPCR) in lung tissue, (2) lung tissue pathology score, and (3) lung radiography score. Correlations were assessed between these outcome measures, the set of signaling features identified in **Figure 4B**, and the frequencies of the previously described cell populations (**Supplementary Table S2**). Descriptions of these variables are provided in the Methods section.

Overall, the strongest correlations were found between mass cytometry features and viral RNA or pathology scores, while radiography outcomes showed weaker associations. The top correlations between cell population frequencies or signaling features versus viral RNA and pathology scores in LN and PBMC samples are illustrated in the biaxial plots in **Figures 5B, C**; additional correlation values are presented in heatmaps (**Supplementary Figures S3A, B**). Notably, correlations consistently trended in the same direction for all clinical outcomes, even when below the statistical significance cutoffs.

Among the most striking findings, PMA/I-triggered CREB phosphorylation in CD56+CD16- NK cells in blood and IκB downregulation in IgM+ B cells in LN were correlated with pathology scores and viral RNA shedding, respectively (**Figure 5C**), suggesting that the severity of SARS-CoV-2 infection is associated with *ex vivo* signaling capacity. Furthermore, the frequency of activated CD8+ T cells (HLA-DR+Ki67+) in PBMCs showed a strong correlation with pathology scores, potentially linking cytotoxic T cell activation — whether as a cause or effect — to viral-mediated pathology. Lastly, the abundance of inflammatory intermediate monocytes in LN was strongly correlated with viral shedding, indicating the recruitment of this monocyte subset to the LN may be proportionate to viral load.

## Discussion

Using mass cytometry to analyze PBMCs and LN cells from rhesus macaques that received either mRNA- or protein-based SARS-CoV-2 vaccines—or no vaccine—followed by a heterologous Delta variant SARS-CoV-2 challenge, we identified notable tissue-specific differences in immune cell frequencies and signaling capacities associated with vaccination. This represents a single timepoint snapshot of immune activity, rather than a longitudinal assessment of the full response trajectory.

Vaccinated animals exhibited reduced frequencies of activated CD8+ T cells in the PBMCs and lower monocyte and B cell subsets in the LN compared to mock-vaccinated controls, mirroring their reduced viral loads and milder pathology. Correlation analyses linked elevated intermediate monocytes in the LN and increased activated CD8+ T cells in the PBMCs with higher viral shedding and more severe pathology. These findings underscore how shifts in immune cell population frequencies align with clinical outcomes and reinforce the protective role of vaccination. These data are consistent with findings of elevated circulating intermediate/inflammatory monocytes in humans with severe COVID - 19 (35) and influenza A infection (17). They also align with previous observations in macaques showing increased frequencies of CD8+ T cells post-challenge (36) and with depletion studies that underscore the critical role of CD8+ T cells in SARS-CoV-2 immunity (11, 37).

*Ex vivo* stimulation (with PMA/I or R848) revealed tempered inflammatory signaling in vaccinated animals, particularly in PBMC NK cells and LN B cells, suggesting that vaccination results in attenuated inflammatory pathways post-challenge. These responses were also correlated with better clinical outcomes. Observations such as elevated PMA/I-triggered pCREB in NK cells and exaggerated R848-triggered IκB downregulation in B cells from unvaccinated animals could reflect general cytokine exposure, metabolic stress, or non-specific immune activation, or alternatively could be mechanistically linked to pathology. Additional investigations, including deeper assessment of B cell subsets, activation states, and functional outputs, will be needed to clarify these possibilities. Although mechanistic studies and validation in additional cohorts is warranted to understand the extensibility of these findings, the signaling responses triggered by PMA/I or R848 observed here were consistent with cell-type and stimulus-specific signaling patterns seen previously in peripheral blood from rhesus macaque models (6).

Plasmacytoid dendritic cells (pDCs) were maintained at higher levels in vaccinated animals’ blood post-challenge, aligning with observations of decreased pDCs in severe human COVID - 19 (24, 28–32). These pDC effects, to our knowledge, have not been previously confirmed in NHP models of SARS-CoV-2; however, pDC depletion from the blood has been described in the context of acute simian immunodeficiency virus (SIV) infection in rhesus macaques (38). Furthermore, in the current study, relative to vaccinated animals, pDCs in unvaccinated controls showed elevated pERK1/2 responses to PMA/I stimulation, which may reflect heightened global signaling associated with ongoing infection, but decreased responses to R848 stimulation, potentially indicating TLR desensitization or redistribution of TLR-sensitized pDCs to infected tissues. Further investigation to clarify how these dynamics enhance or hinder viral control in humans and NHP models may be warranted. Such insights could inform targeted therapeutic strategies to expedite viral clearance.

Both vaccines conferred protection in this study, with the largest differences in immune cell responses observed between mock controls and vaccinated groups, likely reflecting reduced viral control in the mock group rather than direct vaccine effects. The mRNA and protein vaccines elicited largely comparable cellular responses at 7 days post-challenge, with no statistically significant differences after multiple hypothesis correction (**Supplementary Table S2**). The mass cytometry platform may lack the resolution to detect subtle, platform-specific effects, and the single time point analyzed may have been too late to capture protective responses occurring immediately post-challenge. Future studies with earlier and longitudinal sampling will be necessary to determine whether these platforms confer protection through similar or distinct immune pathways.

Several studies (39–42) indicate that circulating immune cells may not fully reflect the composition and function of tissue-resident populations. In humans, Ramirez et al. recently demonstrated that adenoids harbor mucosal immune memory not apparent in blood (43), underscoring the importance of local lymphoid tissues in respiratory immunity. Lymph node responses may indeed provide a more relevant comparison to mucosal immunity than PBMCs alone. In our study, analysis of lung-draining mediastinal lymph nodes alongside peripheral blood highlights the value of including both compartments; however, we did not have the tools to assess antigen specificity of lymph node cells, which will be an important direction for future work. This study underscores the potential benefit of both mRNA- and protein-based SARS-CoV-2 vaccines in reducing disease severity in infant-immunized populations, evidenced by previously reported measurable reductions in clinical outcome severity measures (13), and a reduction in cellular responses associated with these clinical outcome severity measures we observed here. These cellular features could serve as an additional set of biomarkers to help gauge reductions in disease severity in future NHP model studies.

This study has limitations. Although a large number of animals were sacrificed for this study, the sample size (n=8 per vaccine group) may be too small to detect subtle differences that could emerge with larger cohorts. While cell populations were compared based on frequency, direct assessment of the cellularity of either blood or lymph node was not possible. Because we assessed samples only at 7 days post-challenge, any pre-existing differences between groups present immediately before challenge may have been missed. For instance, we cannot rule out whether observed differences between groups pre-existed prior to challenge due to memory/trained immunity from immunization or were a consequence of response to viral challenge. The study also evaluated responses at a single time point approximately one year after vaccination, which may limit the detection of earlier or later immune features relevant to protection. Future studies should more closely examine the link between early and late post-vaccination immune responses and those observed in this study after challenge. While *ex vivo* stimulation assays are informative, they do not necessarily fully recapitulate the capacity of immune cells to respond to stimuli *in vivo.* Furthermore, lymph node cell dissociation disrupts spatial context, potentially obscuring additional insights into cellular responses.

This study used a broad mass cytometry approach to assess immune cell frequency and signaling capacity across different tissues after viral challenge in a key non-human primate model for vaccine development. Future research combining this methodology with longitudinal profiling could uncover early post-viral challenge features related to effective memory responses to be optimized in next-generation vaccine design. Applying the same strategy at early post-vaccination time points may also identify correlates of protection for future immunization approaches. Notably, differences in stimulation-dependent cell signaling responses, which correlated here with vaccination status and clinical severity, remain underexplored as potential predictive biomarkers for disease course, correlates of vaccine-conferred protection, and potential targets for intervention approaches.

## Data Availability

The datasets presented in this study can be found in online repositories. The names of the repository/repositories and accession number(s) can be found in the article/**Supplementary Material**. Unique CyTOF reagents will be made available to the scientific community upon reasonable request by contacting Dr. David McIlwain. Mass cytometry files from this study (.fcs) are available from Mendeley (https://data.mendeley.com/preview/w7685x343j?a=0e265441-8196-4730-b566-bdb6b6111e16). Code used in this study is available from Github (https://github.com/sasha-anronikov/CIV-CyTOF). Any additional information required to reanalyze the data reported in this work paper is available from the lead contact upon request.

## Supplementary material

Supplementary Figure 1 Gating strategy. Hierarchical gating strategy used to annotate cell populations based on phenotypic markers. Parent populations appear above biaxial plots, annotated populations are labeled next to respective gates. Representative plots for a peripheral blood **(A)** and lymph node **(B)** sample. Gating was performed using CellEngine.

Supplementary Figure 2
**(A-D)** Repeated control samples from two animals (38956 and 39142) were barcoded, pooled, stained, and run alongside study samples in multiple batches to assess technical consistency. **(A, B)** For each animal, UMAP plots colored by replicate number show similar distributions for pooled cells **(A)** with those from up to seven replicates **(B)**. **(C,D)** For each animal, UMAP plots colored by annotated cell populations show similar distributions of cell populations between pooled cells **(C)** with those from up to seven replicates **(D)**. **(E)** Frequencies of immune cell populations are consistent between samples treated with the different *ex vivo* stimulations P (PMA/Ionomycin), R (R848), U (unstimulated PBS control).

Supplementary Figure 3 Alluvial plot showing distribution of cell populations across tissues and vaccination groups. The alluvial plot shows the distribution of LN and PBMCs frequencies (axis 1) across vaccine groups (Mock, mRNA, and Protein; axis 2) and immune cell populations (axis 3). Cells originating from LN or PBMCs retain their respective colors throughout the flow. Cell population frequencies are displayed as the mean proportion of mononuclear cells (% of CD45⁺CD66⁻ cells). Each flow represents a cell population, and its width corresponds to the mean frequency of that population. Flows connect tissue type (LN or PBMC), vaccine group, and immune cell identity, allowing for comparison of relative population abundance across compartments.

Supplementary Figure 4 Normalized R848 signaling responses in pDCs. The heatmap shows the R848-induced signaling responses in pDCs in both LN and PBMCs. The color scale indicates the normalized signaling response median channel intensity (stimulated – unstimulated), with yellow representing a positive change (upregulated), black representing a zero value (no change), and purple representing a negative change (downregulated) post stimulation. Data are shown for all animals pooled, as well as separated by vaccination group (Mock, mRNA, and Protein).

Supplementary Figure 5 Heatmaps for additional correlations between immune cell frequencies or signaling pathways, and clinical outcomes. **(A, B)** Heatmaps illustrating correlations between cell population frequencies **(A)** or top cell signaling features **(B)** and clinical outcomes. The color scale represents Pearson’s R values; crosshatching indicates an adjusted p-value > 0.05. Cell frequencies are given as the percentage of mononuclear cells (CD45⁺CD66⁻). Cell signaling is shown as the arcsinh ratio of signaling marker levels relative to unstimulated controls for the indicated cell populations and stimulation conditions (PMA/Ionomycin or R848). Pathology scores are lung tissue pathology scored from 0 to 4. SARS-CoV-2 RNA is measured as N gene (log₁₀ copies per 30 mg of lung tissue), and Radiography score are radiologist’s assessment of lung pathology, scored from 1 to 4.

Supplementary Table 1 Mass cytometry antibody panel. Target antigen, clone, vendor/catalog number, isotope label, and staining concentration are indicated for surface, intracellular, and intracellular signaling markers.

Supplementary Table 2 Cell population frequencies, signaling marker levels, clinical outcome data, and statistics. Data associated with Figures 2-5. Tabs indicate naming conventions; animal-level data for cell population abundance (frequency and ‘pseudo-absolute count’ derived from CBC data), cell population signaling marker levels, and clinical outcomes; statistical comparisons of cell abundance and cell signaling between vaccine groups, and correlation to clinical outcomes.
