# Supplementary material for: Comparative single-cell immune responses in peripheral blood and lymph node of immunized SARS-CoV-2 challenged infant rhesus macaques
Source: Front Immunol. 2025 Sep 15;16:1599408. doi: 10.3389/fimmu.2025.1599408 (PMC12477255; doi:10.3389/fimmu.2025.1599408)
Supplement: Supplementary Figure 1 — Gating strategy. Hierarchical gating strategy used to annotate cell populations based on phenotypic markers. Parent populations appear above biaxial plots, annotated populations are labeled next to respective gates. Representative plots for a peripheral blood (A) and lymph node (B) sample. Gating was performed using CellEngine. [file Supplementaryfile1.pdf]

A.

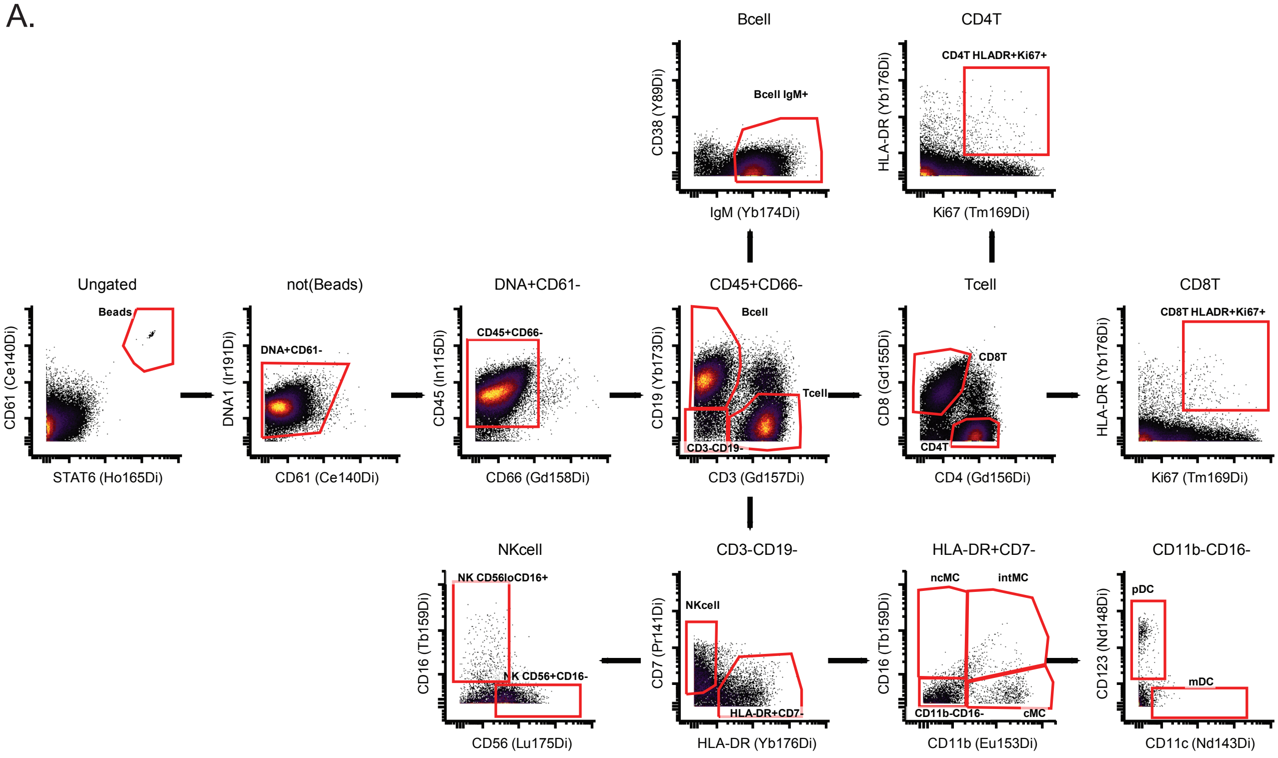

B.

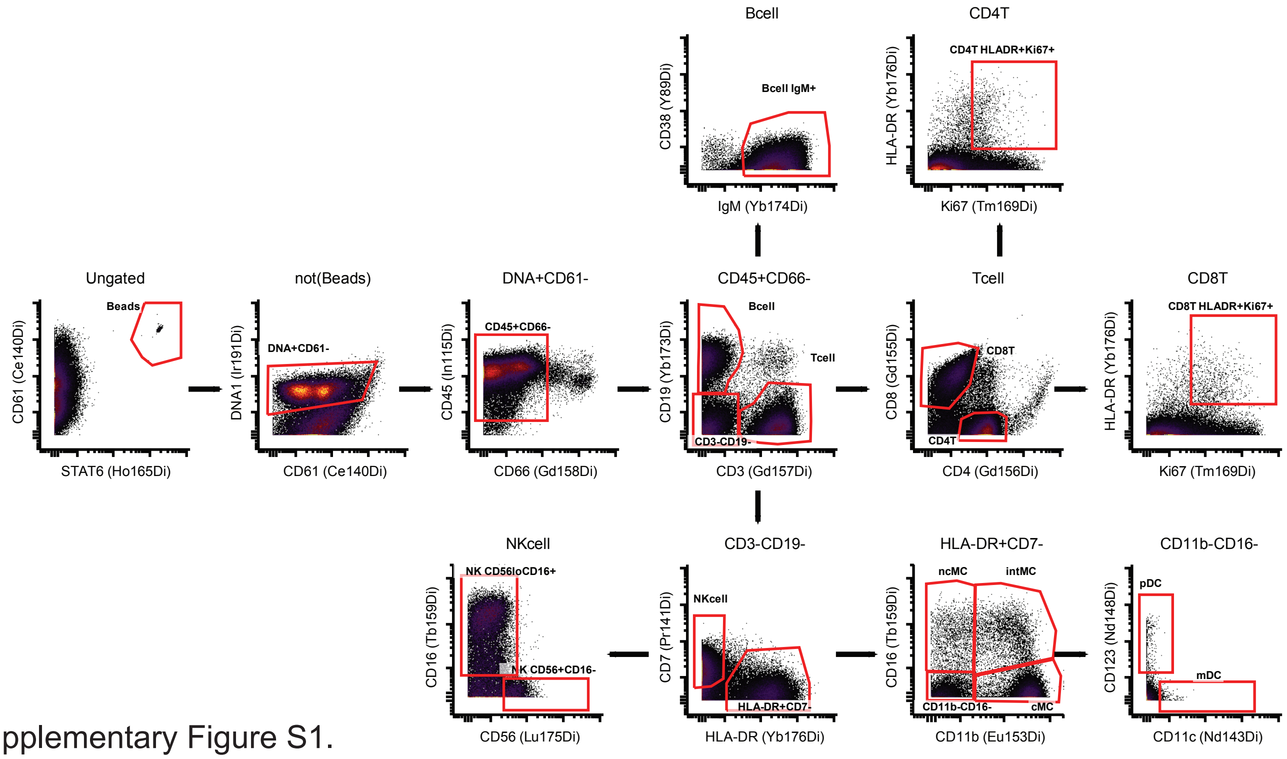

Supplementary Figure S1.

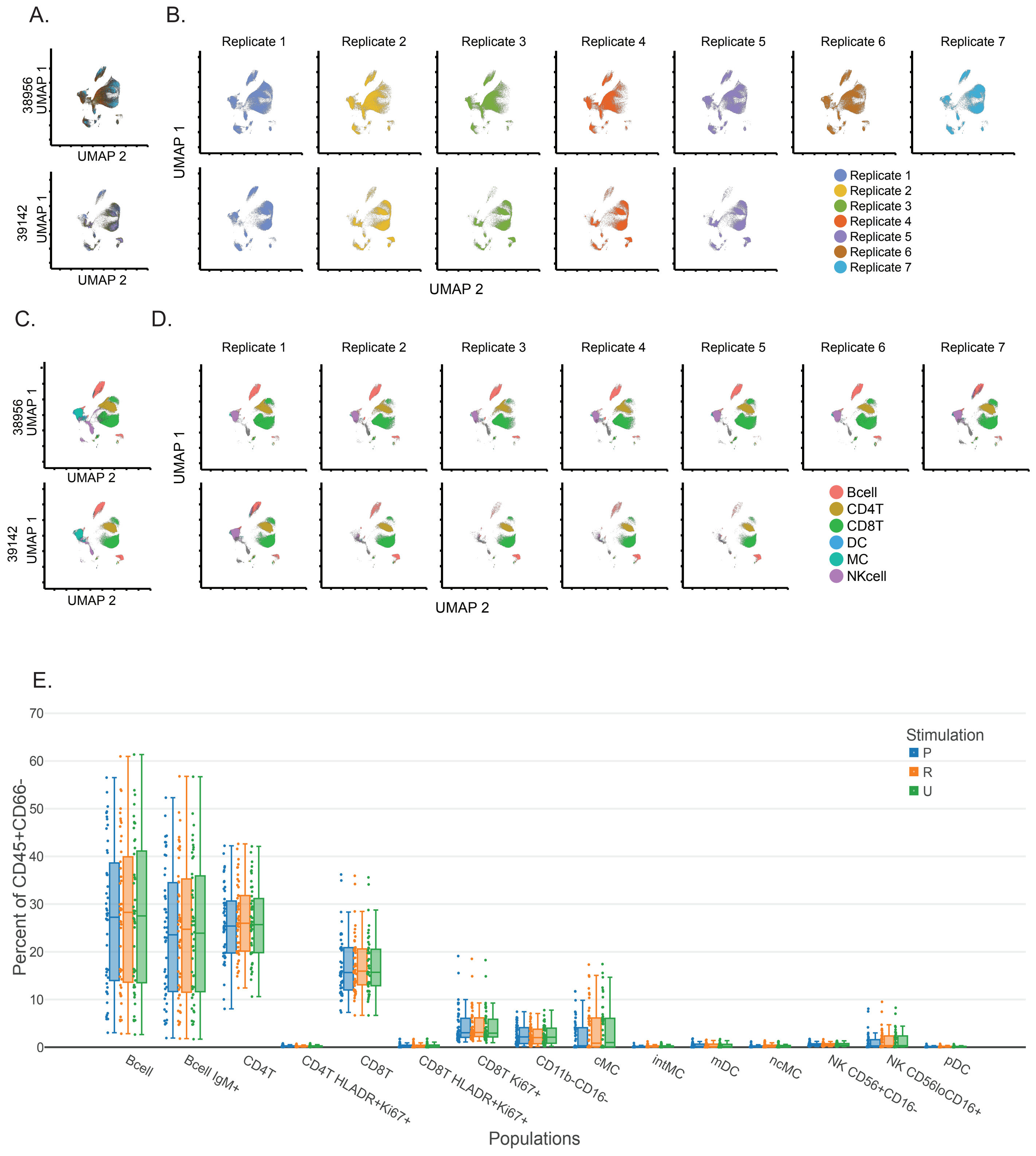

Supplementary Figure S2.

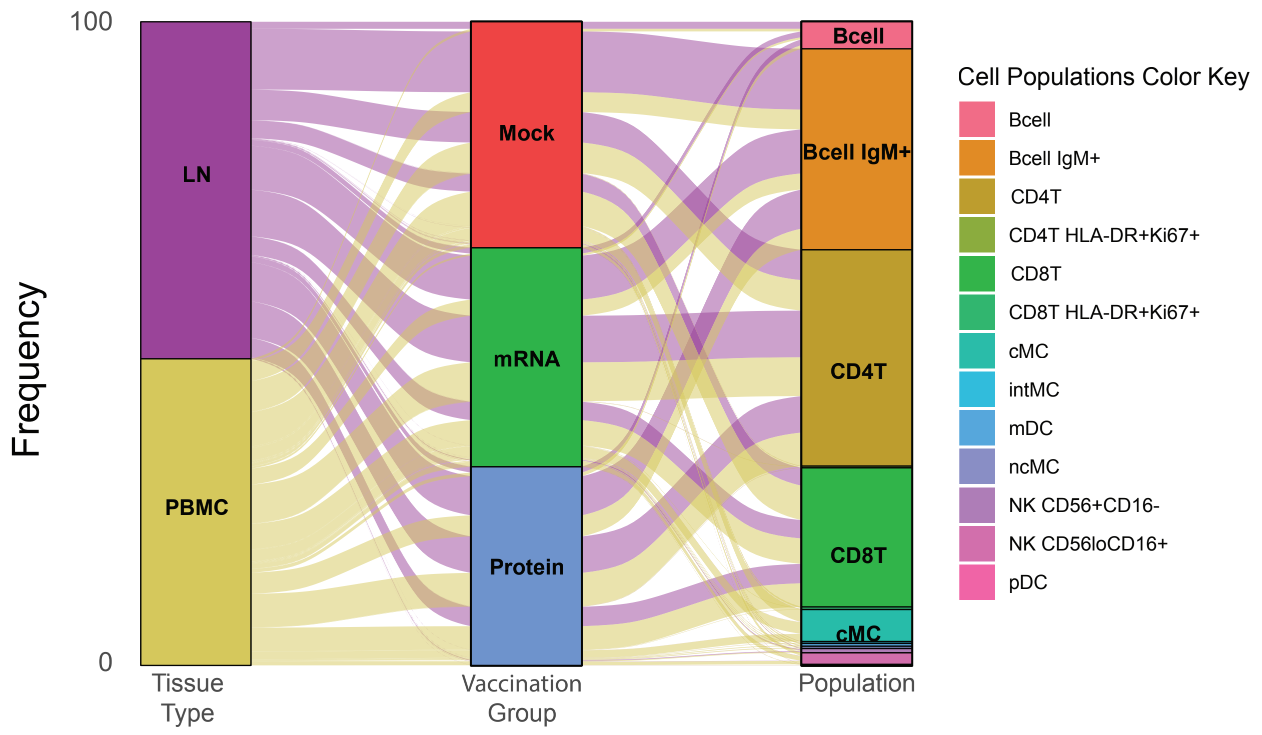

Supplementary Figure S3.

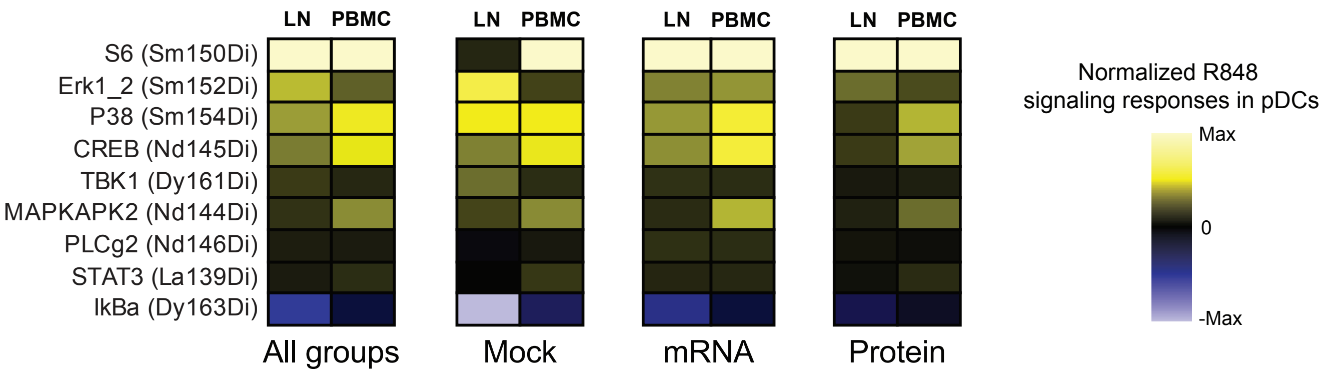

Supplementary Figure S4.

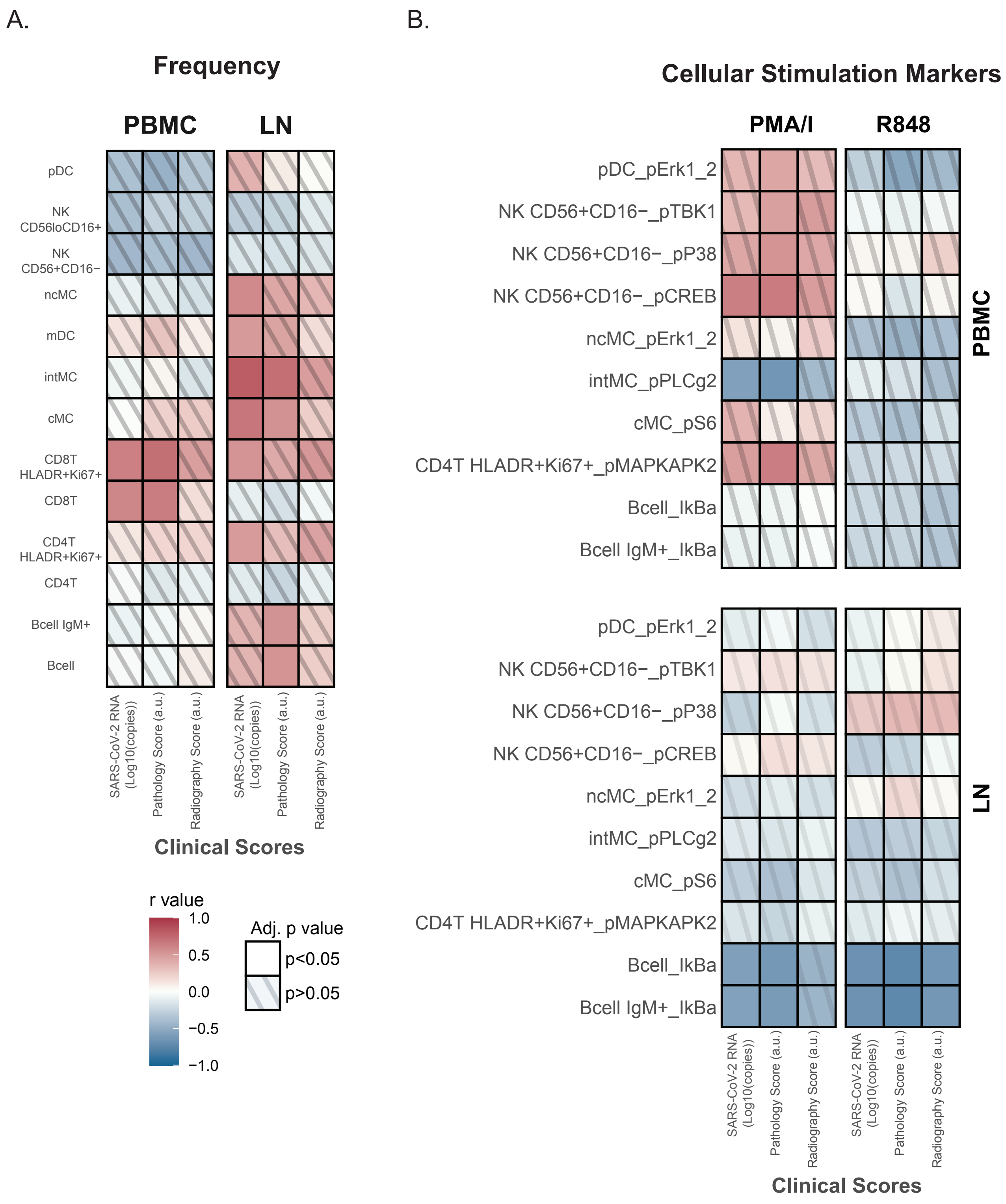

Supplementary Figure S5.

Supplemental Table 1. Mass cytometry antibody panel.

Antigen	Clone	Isotope Label	Vendor and Catalog	Straining Concentration (µg/ml)	Stain type
CD233	BRIC 6	113	IBGRL 9439	4.1	Surface
CD45	DO58-1283	115	BD 552566	5.4	Surface
CD61	VI-PL2	140	Biolegend 336402	5.4	Surface
CD7	M-T701	141	BD 555359	5.4	Surface
CD33	AC104.3E3	142	Miltenyi	4.3	Surface
CD11c	3.9	143	Biolegend	1.3	Surface
CD123	7G3	148	BD 554527	1.3	Surface
CD14	M5E2	151	Biolegend 301810	10.7	Surface
CD11b	ICRF44	153	Biolegend 301312	6.7	Surface
CD8	RPA-T8	155	Biolegend 301002	1.4	Surface
CD4*	OKT4	156	Biolegend 317404	1.3	Surface
CD3	SP34.2	157	BD 551916	2.7	Surface
CD66	YTH71.3	158	Thermo MA5-17003	2.3	Surface
CD16	3G8	159	Biolegend 302033	2.7	Surface
CD1c	AD5-8E7	162	Miltenyi	1.3	Surface
BDCA3	1A4	164	BD 559780	3.6	Surface
CD45RA	HI100	166	Biolegend 304102	2.7	Surface
CD161	HP-G310	168	Biolegend 339902	3.1	Surface
FITC	FIT-22	171	Biolegend 408302	5.4	Surface
CD20	2H7	173	Biolegend 302302	5.4	Surface
IgM	G20-127	174	BD 555780	2.7	Surface
CD56	NCAM16.2	175	BD 559403	1.8	Surface
HLA-DR	Immu357	176	Beckman Coulter	0.3	Surface
CCR7	150503	FITC	BD 561271	8.9	Surface
STAT1 pY701	4a	147	BD Biosciences 612233	3.8	Intracellular (signaling marker)
STAT3 pY705	4	139	BD Biosciences 612357	5.8	Intracellular (signaling marker)
STAT4 pY693	38	170	BD Biosciences 612738	5.7	Intracellular (signaling marker)
STAT5 pY694	46	149	BD Biosciences 611965	5.4	Intracellular (signaling marker)
STAT6 pY691	18, J71-773.58.11	165	BD Biosciences 611597	5.4	Intracellular (signaling marker)
Ki67	SolA15	169	Thermo Fisher 14-5698-82	3.4	Intracellular
Erk1/2 pT202/Y204	D13.14.4E	152	CST 4370	16.4	Intracellular (signaling marker)
MAPKAPK2 pT334	27B7	144	CST 3007	2.3	Intracellular (signaling marker)
CREB pS133	87G3	145	CST 9198	7.2	Intracellular (signaling marker)
IkBa amino-terminal	L35A5	163	CST 4814	3.8	Intracellular (signaling marker)
TBK1/NAK pS172	D52C2	161	CST 5483	10.8	Intracellular (signaling marker)
S6 pS235/236	2F9	150	CST 4856	13.2	Intracellular (signaling marker)
Zap70/Syk pY319/Y352	17a	160	BD Biosciences 612574	2.1	Intracellular (signaling marker)
4E-BP1 pT37/46	236B4	172	CST 2855	3.1	Intracellular (signaling marker)
PLCγ2 pY759	K86-689.37	146	BD Biosciences (Custom)	2.2	Intracellular (signaling marker)
P38 pT180/Y182	36/p38	154	BD Biosciences 612289	3.6	Intracellular (signaling marker)
FITC (for CCR7)	FIT-22	171	Biolegend 408302	5.3	Intracellular
FoxP3	PCH101, NRRF-30	167	Thermo Fisher 14-4776-82	10.6	Intracellular

Supplementary Table S1.

For Supplementary Table S2 see Table 1.xlsx

Supplementary Table S2.
